# Simultaneous ST-Elevation Myocardial Infarction and Ischemic Stroke: A Therapeutic Dilemma and a Case for Deferred Revascularization

**DOI:** 10.7759/cureus.104265

**Published:** 2026-02-25

**Authors:** Youssef Daoudi, Lebbar Samy, Hibat Allah Kamri, Hajar Rabii, Fatimazahra Merzouk, El Ghali Mohamed Benouna

**Affiliations:** 1 Cardiology, Cheikh Khalifa Ibn Zaid International University Hospital, Casablanca, MAR; 2 Cardiology, Mohammed VI International University Hospital, Bouskoura, MAR; 3 Cardiovascular Medicine, Mohammed VI International University Hospital, Bouskoura, MAR; 4 Cardiology, Cheikh Khalifa International University Hospital, Mohammed VI University of Health Sciences (UM6SS), Casablanca, MAR; 5 Cardiology, Mohammed VI International University Hospital, Mohammed VI University of Health Sciences (UM6SS), Casablanca, MAR

**Keywords:** cardio-neurology, dual pathology, ischemic stroke, percutaneous coronary intervention, stemi, temporized angiography

## Abstract

The simultaneous occurrence of acute ischemic stroke and ST-elevation myocardial infarction (STEMI), termed cardio-cerebral infarction (CCI), represents a rare and challenging emergency due to competing therapeutic priorities. No clear guidelines currently exist to guide management in such cases. A 63-year-old male with uncontrolled hypertension presented with 48 hours of progressive dyspnea and epigastric discomfort, followed by sudden-onset left hemiplegia and dysarthria. On admission, brain CT revealed a right middle cerebral artery infarction without hemorrhage. ECG showed inferior STEMI, and troponin levels were elevated. Given the high hemorrhagic risk, urgent coronary angiography was deferred. A conservative strategy was adopted with aspirin (75 mg/day) and intermediate-dose low-molecular-weight heparin (enoxaparin 40 mg BID), initiated within six hours of neuroimaging. The patient’s neurological status gradually improved, with National Institutes of Health Stroke Scale scores decreasing from 17 at admission to 10 at discharge, and a modified Rankin Score of 3. One month later, he reported recurrent angina. Coronary angiography revealed spontaneous reperfusion of the right coronary artery, allowing successful percutaneous coronary intervention with three stents. This case illustrates a stroke-first approach based on neurologic severity and hemodynamic stability. Antithrombotic management balanced ischemic and hemorrhagic risks. Timely neurologic improvement allowed deferred but successful revascularization, emphasizing the importance of individualized care in CCI.

## Introduction

The simultaneous occurrence of ST-elevation myocardial infarction (STEMI) and acute ischemic stroke, termed cardio-cerebral infarction (CCI), is a rare but life-threatening condition with an estimated prevalence of 0.009% among ischemic stroke patients [1]. This scenario presents a therapeutic paradox, as the management strategies for each condition, i.e., rapid coronary revascularization for STEMI and cautious cerebral reperfusion for stroke, may be mutually exclusive or even harmful when applied concurrently. For example, antiplatelet or anticoagulant therapies critical for STEMI can exacerbate the risk of hemorrhagic transformation in large cerebral infarcts [2].

Current evidence-based guidelines, including the 2021 American Heart Association/American Stroke Association (AHA/ASA) guidelines for acute ischemic stroke and the 2023 European Society of Cardiology (ESC) guidelines for acute coronary syndromes, provide no specific recommendations for the concurrent management of STEMI and stroke [3,4]. In the absence of consensus, individualized approaches have been reported. A stroke-first strategy is generally favored in patients with large infarcts or high National Institutes of Health Stroke Scale (NIHSS) scores, where the risk of intracerebral bleeding outweighs cardiac urgency. Conversely, a heart-first approach may be justified in unstable patients with cardiogenic shock, ongoing chest pain, or malignant arrhythmias [5].

This report presents a case of CCI managed with conservative medical therapy during the acute neurologic phase, followed by successful deferred coronary angioplasty. The management pathway was chosen based on neurologic severity, absence of hemodynamic instability, and close cardiology-neurology coordination.

## Case presentation

A 63-year-old male (weight: 78 kg) with a history of poorly controlled hypertension and a left pertrochanteric fracture in 2021 presented to a local healthcare facility with a 48-hour history of progressive dyspnea and epigastric discomfort concomitant with left hemiplegia and dysarthria. He was subsequently brought to the emergency department for evaluation and management.

**Figure 1:**
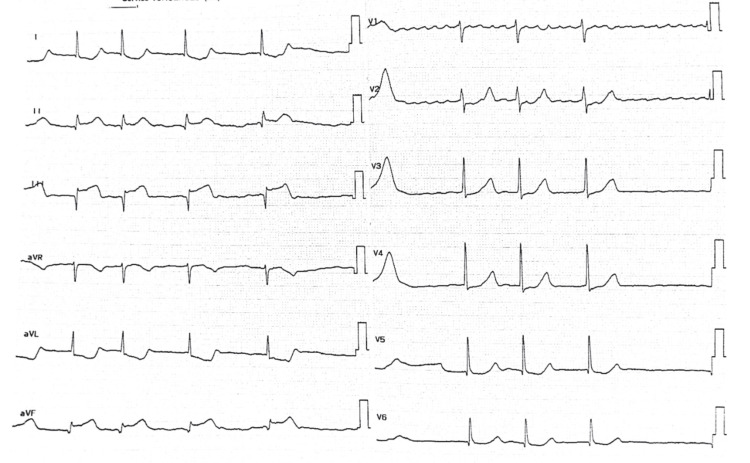
Electrocardiogram showing atrial fibrillation. ST-segment elevation is present in the inferior leads (II, III, aVF).

Upon admission to the intensive care unit, the patient was conscious but confused (Glasgow Coma Scale (GCS) score: 13/15) with left brachial monoplegia, left lower limb paresis (3/5), facial involvement, and dysarthria. Neurological severity was scored at an NIHSS score of 17. Vital signs showed a blood pressure of 130/65 mmHg and bradycardia at 48 beats/minute. Oxygen saturation was 98% on room air. Cardiovascular examination revealed normal heart sounds without murmur. ECG confirmed atrial fibrillation with STEMI in the inferior leads (Figure 1).

Initial laboratory tests showed elevated troponin I (252 ng/L). Additional laboratory investigations revealed leukocytosis (16,920/mm³), C-reactive protein of 67.5 mg/L, hemoglobin level of 15.2 g/dL, platelet count of 169,000/mm³, and a normal coagulation profile (prothrombin time: 93.7%). Renal function was monitored and remained stable during hospitalization.

**Figure 2:**
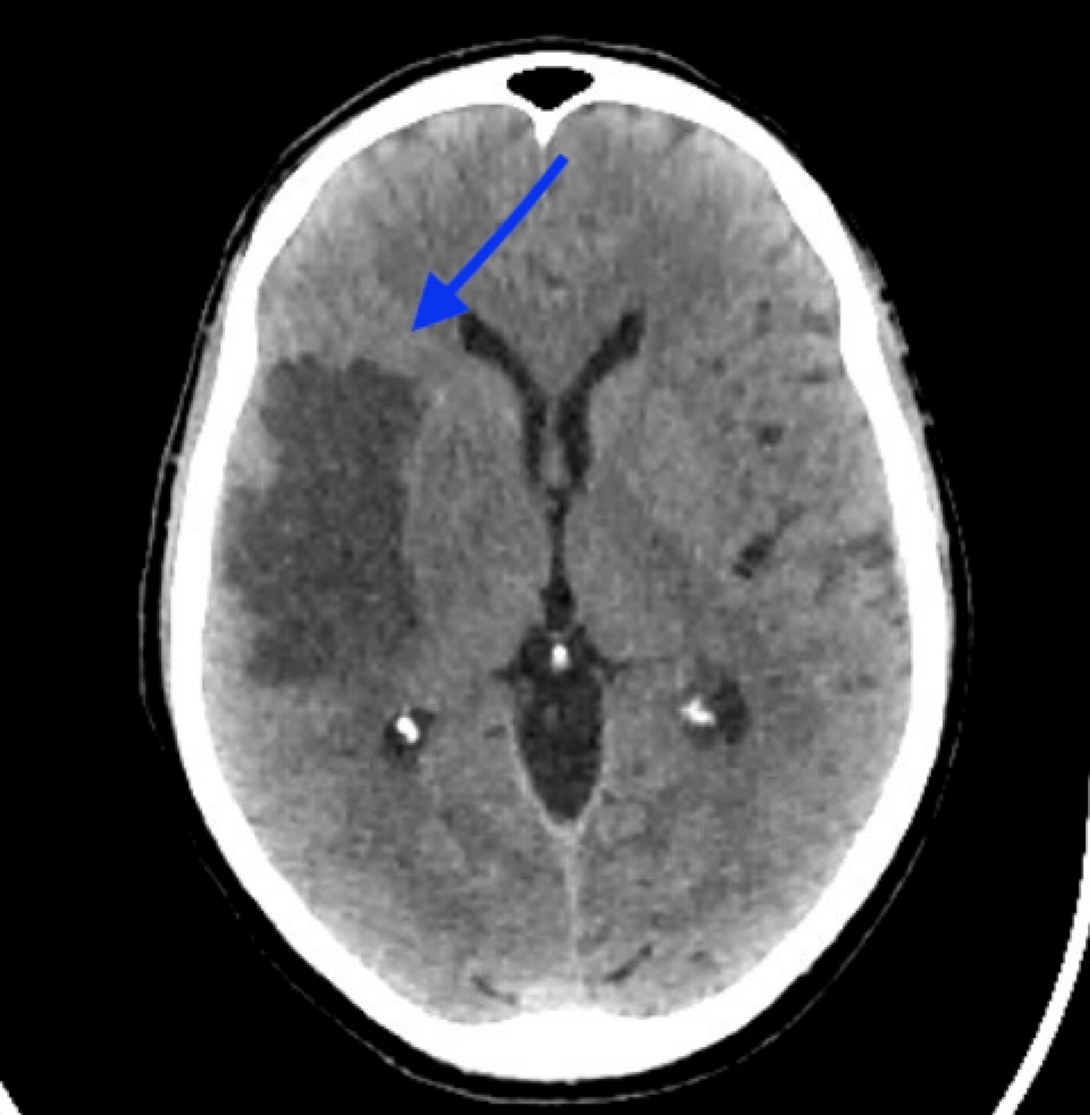
Brain CT scan on admission. Brain CT demonstrating a large right middle cerebral artery territory infarction, with significant hypodensity and loss of grey-white matter differentiation ( blue arrow). These findings are consistent with an acute ischemic stroke involving the right middle cerebral artery territory.

A non-contrast brain CT scan revealed an acute ischemic infarction in the right superficial middle cerebral artery (MCA) territory (Figure 2).

Transthoracic echocardiography demonstrated a preserved left ventricular ejection fraction, minimal mitral and aortic regurgitation, dilated right heart chambers with right ventricular dysfunction, and no evidence of left ventricular apical thrombus.

A comprehensive etiological workup was conducted. Although atrial fibrillation was considered the most probable cause of stroke, a full diagnostic evaluation was performed to exclude alternative mechanisms. Carotid ultrasound demonstrated no significant extracranial carotid stenosis. Neuroimaging did not identify large-vessel atherosclerotic occlusion requiring mechanical thrombectomy. An etiological laboratory panel, including coagulation profile, inflammatory markers, and thrombophilia screening, did not reveal evidence of hypercoagulable disorder or systemic vasculitis. Despite this extensive evaluation, no alternative mechanism was identified, and the stroke was classified as cardioembolic secondary to atrial fibrillation.

Given the significant neurological involvement and high risk of hemorrhagic transformation, a multidisciplinary decision was made to defer urgent coronary angiography. Instead, a conservative medical strategy was adopted with initiation of aspirin (75 mg/day) and intermediate-dose low-molecular-weight heparin (enoxaparin 40 mg BID), introduced within six hours of neuroimaging. Blood pressure was maintained below 140/90 mmHg during the acute phase. Follow-up brain CT on day two revealed a porencephalic cavity without hemorrhagic transformation, consistent with infarct evolution.

The patient’s neurological status progressively improved: he regained full consciousness (GCS score: 15) by day three, with partial recovery of motor function in the left lower limb (4/5), although brachial monoplegia persisted. The NIHSS score decreased from 17 at admission to 13 at day three and 10 at discharge, with a modified Rankin Score (mRS) of 3. Hemodynamically stable throughout hospitalization, the patient was transferred to the cardiology unit for further evaluation.

During the subsequent hospitalization period and before discharge, anticoagulation was escalated to weight-adjusted, therapeutic-dose low-molecular-weight heparin (enoxaparin 1 mg/kg twice daily), given the confirmed cardioembolic mechanism related to atrial fibrillation with multidisciplinary agreement. Close neurological monitoring was maintained to minimize hemorrhagic risk.

**Figure 3:**
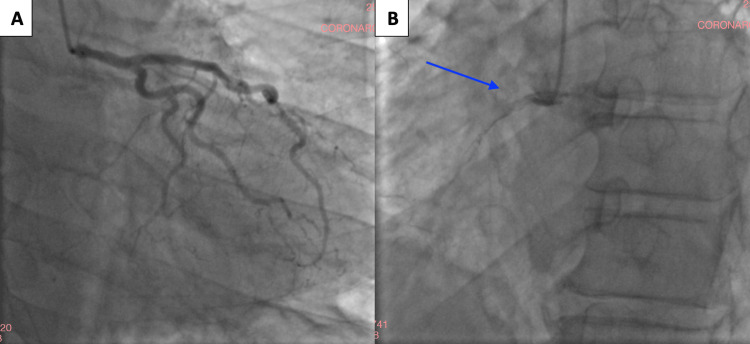
Coronary angiography findings. (A) Left coronary system in the left anterior oblique caudal projection, showing a normal left coronary artery without significant stenosis. (B) Right coronary artery in the right anterior oblique projection, demonstrating a complete proximal occlusion (blue arrow).

The coronary angiogram demonstrated a complete occlusion of the right coronary artery, consistent with the previously suspected culprit lesion (Figure 3).

The patient remained hemodynamically stable throughout hospitalization and was subsequently discharged home after neurological stabilization and initiation of medical therapy. He was followed on an outpatient basis with clinical monitoring and continued neurological rehabilitation.

**Figure 4:**
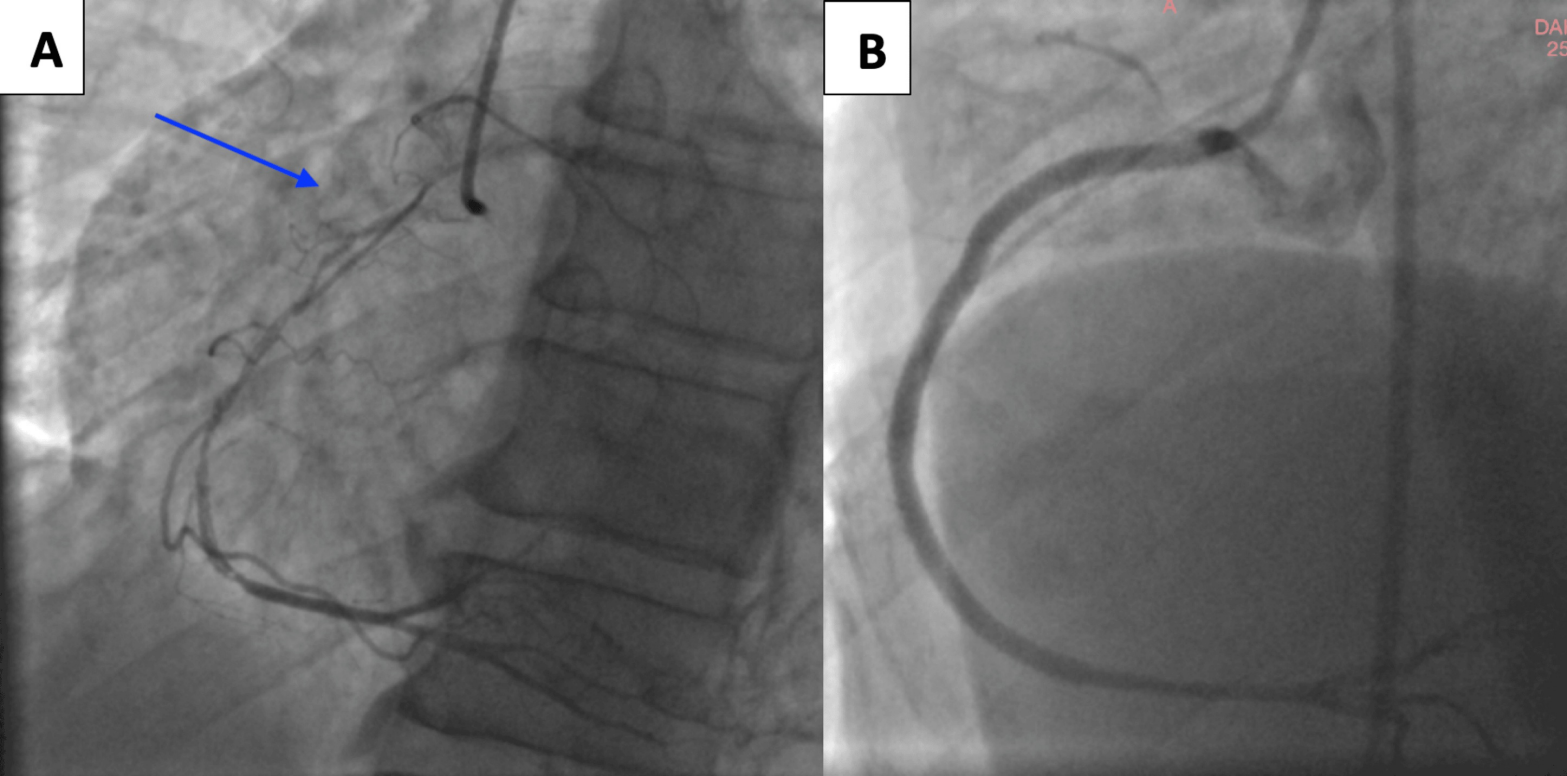
Coronary angiography and final result after percutaneous coronary intervention. (A) Right coronary artery in the right anterior oblique cranial projection, showing spontaneous reperfusion of the previously occluded segment (blue arrow). (B) Final angiographic result in the right anterior oblique projection after successful percutaneous coronary intervention with implantation of three stents, restoring Thrombolysis in Myocardial Infarction 3 flow.

One month after the initial event, the patient reported recurrent angina. A coronary angiography revealed spontaneous reperfusion of the previously occluded right coronary artery. This favorable evolution enabled successful percutaneous coronary intervention (PCI) of the right coronary artery, which revealed isolated single-vessel disease. The procedure was technically challenging due to the presence of resistant atherosclerotic plaques and initial difficulty in advancing the guidewire across the lesion. However, successful crossing and stent deployment were ultimately achieved, restoring optimal coronary flow (Figure 4).

At discharge, and following neurological stabilization, oral anticoagulation with apixaban 5 mg BID was initiated in agreement with the neurology team. Given the recent PCI and the presence of atrial fibrillation, a triple antithrombotic therapy was prescribed initially, combining apixaban, aspirin (75 mg/day), and clopidogrel (75 mg/day). This regimen was maintained for only one week, after which aspirin was discontinued to minimize bleeding risk, and dual therapy with apixaban and clopidogrel was continued. This strategy aligns with current ESC guidelines for patients at high bleeding risk requiring anticoagulation and recent PCI. The patient was also discharged on a beta-blocker, high-dose statin, and supportive neurologic rehabilitation therapy.

## Discussion

The simultaneous occurrence of acute ischemic stroke and STEMI, often referred to as CCI, is an extremely rare clinical entity that presents a formidable therapeutic challenge due to competing priorities of urgent coronary revascularization and cerebral reperfusion. Acute myocardial infarction and acute ischemic stroke each have well‑established evidence‑based treatments; however, their concurrence creates a treatment paradox in which therapies for one condition (e.g., antiplatelet agents, anticoagulation, catheter‑based reperfusion) may significantly heighten the risk of hemorrhagic transformation of the cerebral infarct [3].

There is no consensus guideline for managing CCI, largely because of its rarity and the heterogeneous clinical presentations reported in the literature. A recent case report described a 67‑year‑old male with concurrent acute STEMI and right MCA stroke who was treated with tenecteplase followed by mechanical thrombectomy, with a good clinical outcome, illustrating one potential strategy when both stroke and myocardial reperfusion can be addressed emergently in centers with neurointerventional capacity [4].

Conservative neuro‑first strategy: Some cases reported initial treatment of the stroke with intravenous thrombolysis (e.g., tissue plasminogen activator) alone, followed by delayed coronary intervention or supportive care when thrombolysis was considered safe and within the stroke window. However, outcomes in such reports have been mixed, and the risk of myocardial progression remains a concern [5].

Delayed or staged PCI after initial conservative management: Several authors emphasize individualized care based on clinical severity, neurologic deficit, and bleeding risk. A meta‑analysis of 94 cases of concurrent CCI showed that patients treated with combined PCI and mechanical thrombectomy had better short‑term and 90‑day outcomes compared with medical therapy alone, suggesting benefit in centers capable of coordinated intervention [6].

Simultaneous or hybrid approaches: A few case reports describe innovative hybrid strategies, including simultaneous intracoronary and cerebral intra‑arterial thrombolysis or combined PCI with mechanical thrombectomy in a single session under multidisciplinary coordination, though these remain anecdotal and dependent on advanced institutional capabilities [3,4].

In addition, multiple case series, including reports of co‑occurrence with Trousseau syndrome or other hypercoagulable states, highlight the pathophysiologic complexity and need for tailored management [6].

In the present patient, neurologic severity (NIHSS score: 17) and the high risk of hemorrhagic transformation guided an initial conservative medical approach, with antithrombotic therapy optimized to balance cerebral risk and myocardial ischemia. The absence of hemodynamic instability or refractory arrhythmias provided a window to temporize definitive coronary revascularization. This strategy was consistent with some case reports advocating delayed or staged revascularization in situations where immediate invasive strategies pose disproportionate neurologic risk [6].

The subsequent successful PCI one month later supports the feasibility of this approach in selected patients, particularly when neurologic stabilization has been achieved, and the acute phase of cerebral infarction has passed.

Several hypotheses have been proposed to explain concomitant CCI, including simultaneous embolization of cardiac thrombi to cerebral and coronary arteries, atrial fibrillation with multi‑territory embolization, or a primary cerebral infarct triggering cardiac events through autonomic dysregulation. The heterogeneity of mechanisms underscores the importance of thorough diagnostic evaluation [7].

The meta-analysis by Habib et al. [6] defined outcomes based on early neurologic and cardiac recovery, in-hospital mortality, and 90-day mRS, while accounting for case heterogeneity through stratification by treatment modality and initial clinical severity. In our case, atrial fibrillation, documented on ECG but previously undiagnosed, supported a cardioembolic mechanism, favoring early anticoagulation and influencing the choice of a conservative strategy. Combined or hybrid reperfusion approaches were not pursued despite institutional capability, given the absence of large-vessel occlusion, high NIHSS score, and the need to minimize cerebral bleeding risk. Antithrombotic management was escalated gradually: initial aspirin and intermediate-dose low-molecular-weight heparin transitioned to triple therapy (aspirin + clopidogrel + apixaban) post-PCI, then reduced to apixaban plus clopidogrel after one week. This stepwise approach prioritized neurologic safety while maintaining coronary protection.

Given the poor prognosis historically associated with CCI, with mortality rates approximating one‑third at discharge and nearly half by 90 days in pooled case analyses, individualized, multidisciplinary decision-making is vital [6]. Patients presenting in centers with hybrid capabilities may benefit from coordinated PCI and neurointerventional approaches. Conversely, patients with large cerebral infarcts or prohibitively high bleeding risk may be best managed initially with conservative medical therapy and staged revascularization, as illustrated in this case.

Further research should prioritize the creation of prospective registries, standardization of case reporting elements, and development of consensus pathways for CCI management, especially concerning the timing and safety of antithrombotic therapies in the acute dual-injury setting.

## Conclusions

This case highlights the feasibility of a conservative, neuro-prioritized approach in managing concurrent acute ischemic stroke and STEMI in selected patients. Given the absence of hemodynamic instability, the high risk of hemorrhagic transformation, and evidence of cardioembolic stroke from new-onset atrial fibrillation, delayed revascularization proved both safe and effective after neurological stabilization. While this management strategy remains hypothesis-generating rather than practice-informing, it may be considered in patients presenting with large cerebral infarcts, high NIHSS scores, or high bleeding risk. Key selection criteria for delayed PCI could include stable hemodynamics, absence of malignant arrhythmias, and lack of large-vessel coronary occlusion causing ongoing ischemia. Moving forward, prospective registries, standardized case definitions, and treatment algorithms are essential to guide evidence-based decision-making in this rare but high-stakes scenario.
